# Editorial for the Specific Issue: “Lipid-Based Nanocarriers”

**DOI:** 10.3390/biomedicines10071734

**Published:** 2022-07-19

**Authors:** Chin-Tin Chen

**Affiliations:** Department of Biochemical Science and Technology, National Taiwan University, No. 1, Sec. 4, Roosevelt Road, Taipei 10617, Taiwan; chintin@ntu.edu.tw

Small molecules and biologics are the two major categories of active pharmaceutical ingredients (APIs) commonly used for disease management. Lipid-based nanocarriers have been shown to protect APIs from biological degradation and to modify their bioavailability, allowing for their more efficient delivery to the target tissue while reducing toxicity to other organs [1]. Presently, liposomal drugs used in oncology are the most successful lipid nanocarriers in the pharmaceutical market. Lipid nanoparticles (LNPs) are regarded as a new generation of lipid nanocarriers. They have a more complex internal lipid architecture with a low or minimal internal aqueous core. The recent COVID-19 mRNA vaccines were made possible in part due to LNP development, which not only efficiently express the mRNA encoded antigen but also acts as an adjuvant in vaccine reactogenicity [2]. The success of Onpattro^®^ and COVID-19 mRNA vaccines has demonstrated the feasibility of using RNA therapeutics to treat many serious and life-threatening diseases. Developments in molecular medicine have identified the causative mutations of genes involved in diseases’ pathophysiology, providing previously undruggable targets for disease management using RNA therapeutics. In this Specific Issue, two review articles by Lee et al. [3] and Su et al. [4] illustrate the possible applications of RNA-based therapeutics in managing single-gene neurological disorders and chronic kidney diseases, respectively.

Recently, herbal medicines have been extensively investigated for their therapeutic potential against microbial infection and anti-inflammatory effects. However, like most small molecules, these phytochemicals face the problems of poor solubility and bioavailability, which restrict their clinical applications. A liposomal formulation composed of phosphatidylcholine and cholesterol developed by Allemailem et al. was used to encapsulate the main constituent of *Nigella sativa*, thymoquinone, and its therapeutic potential was investigated in vitro and in vivo by treating *A. baumannii*-induced pneumonia in leukopenia mice [5]. This study indicates the promise of using thymoquinone-loaded liposome to treat *A. baumannii* infection and its associated complications.

Enhanced permeability and retention effects have been regarded as the mechanisms by which lipid nanocarriers can passively accumulate in tumors. However, the blood–brain barrier remains the major challenge for delivering nanocarriers to the brain. In many animal studies, the intranasal route has been shown to be an alternative approach to bypass the blood–brain barrier to deliver drugs to the central nervous system without prior absorption to the circulating blood. In this Specific Issue, Lamptey et al. developed multifunctional fatty acid grafted polymeric micelles to deliver VGF to the brain through the intranasal route [6]. This gene delivery system is not a typical lipid-based nanocarrier. However, chitosan modified with oleic acid can enhance their adsorption onto the lipophilic cellular membranes, which increases the gene transfection efficacy.

Whether liposomal drugs can be used for clinical purposes depends on the nanoparticle stability and encapsulation efficiency of drugs in liposomes. Remote loading driven by ion gradient or a transmembrane pH across the liposomes is used to load doxorubicin and irinotecan into PEGylated liposomes and to prevent drugs from being expelled from the nanocarriers. However, the poor release of drugs from liposomes might hinder the therapeutic efficacy. The conventional approach to formulation development is based on the use of routine excipients and simple optimization practices, which usually study one variable at a time and keep all other variables constant. In this specific issue, Lee et al. develop a Doxorubicin-loaded lipid nanocarrier based on electrostatic interactions using bottom-up microfluidic technology [7]. Using quality by design, the formulation was optimized for loading doxorubicin into the lipid nanocarrier. The resulting dox-loaded lipid nanocarriers not only have a high drug-to-lipid ratio but also exhibit the property of preferred release in acidic environments, which could potentially overcome the poor drug release of the marketed liposomal drug. This approach establishes significant parameters for the more rational design of loading small molecules into nanocarriers that can overcome the complexities and difficulties of multi-step procedures for the development of conventional liposomal drugs.

One strategy for site-specific drug release is to utilize the unique pathophysiological features of diseased tissue to destabilize the nanocarrier, thereby enhancing drug release at the target tissue. It has been shown that elevated acid sphingomyelinase activity plays a role in inflammatory bowel disease. Based on this principle, Penate Medina et al. developed a liposomal delivery vesicle containing sphingomyelin (SM), which can be degraded by sphingomyelinase, resulting in liposomal leakage [8]. The feasibility of using this SM-liposome for bowel inflammation imaging and targeting was verified in vitro and in an acute colitis mouse model. This study demonstrates the feasibility of developing a targeting vehicle for diseased tissue, which could be further used to deliver a wide variety of drugs in the future.
